# Retraction: Alpha-Fetoprotein Promoter-Driven Cre/LoxP-Switched RNA Interference for Hepatocellular Carcinoma Tissue-Specific Target Therapy

**DOI:** 10.1371/journal.pone.0295656

**Published:** 2023-12-05

**Authors:** The PLOS ONE Editors

Following the publication of this article [1], concerns were raised regarding Figs 2, 3, and 4. Specifically:

- In Fig 2C, lanes 2 and 6 in the left Beclin 1 panel appear similar.
- In Fig 2C, the left and right GAPDH panels appear similar despite representing different conditions.
- In Fig 4A Beclin 1 panel, when levels are adjusted to visualize the background, there appears to be vertical discontinuity between lanes 1 and 2.
- In the Beclin 1 panel in Fig 4C, when levels are adjusted, there are similar patterns in the background above the bands in lanes 1 and 5. In addition, in lane 5, the signal around the band appears to be discontinuous with the overall background of the panel.
- The L-02 cell line used as a control for healthy human hepatic tissue was found to be potentially contaminated by HeLa cells in a later study [2].

The authors stated that the original, underlying data for the Western blots in this article are no longer available; however, they provided versions of the images that were cropped wider than the published panels.

Regarding the concerns in Fig 2, the authors stated that the bands in lanes 2 and 6 were similar but were not identical. The authors stated that the similarities observed in the GAPDH panel were caused by an error during figure preparation and provided a replacement panel. In the absence of raw data, the *PLOS ONE* Editors remain concerned about this figure.

The authors stated the vertical discontinuity in Fig 4A may be caused by a smear on the Western blot film. In the absence of original image data, these issues cannot be clarified.

Regarding the Beclin 1 panel in Fig 4C, several repeating patterns were observed in the background of the uncropped panel provided by the authors, in addition to the concerns raised in lanes 1 and 5 of the published panel. The authors stated that the discontinuous background was potentially caused by blemishes on the original film. The *PLOS ONE* Editors remain concerned about the issues involving this figure.

The authors stated that they were not aware of the potential contamination issues with the L-02 cell line at the time of this study, and they no longer have a sample of these cells to investigate. They commented that the morphology of L-02 and HeLa cells in this study was not similar as shown in Figs 1 and 3C of [1].

In light of the concerns affecting multiple figure panels that question the reliability of these data, the *PLOS ONE* Editors retract this article.

YFP did not agree with the retraction. YHS, ZBD, JZ, SJQ, BH, CYG. HY, WRL, and JF either did not respond directly or could not be reached.

## References

[1] Peng Y-F, Shi Y-H, Ding Z-B, Zhou J, Qiu S-J, Hui B, et al. (2013) Alpha-Fetoprotein Promoter-Driven Cre/LoxP-Switched RNA Interference for Hepatocellular Carcinoma Tissue-Specific Target Therapy. PLoS ONE 8(2): e53072. 10.1371/journal.pone.0053072 23468839 PMC3585287

[2] Ye F, Chen C, Qin J, Liu J, Zheng C. Genetic profiling reveals an alarming rate of cross-contamination among human cell lines used in China. FASEB J. 2015 Oct;29(10):4268–72. doi: 10.1096/fj.14-266718. Epub 2015 Jun 26. . 26116706

